# The current status and trend of clinical pharmacology in developing countries

**DOI:** 10.1186/2050-6511-14-49

**Published:** 2013-09-28

**Authors:** Andrew Walubo

**Affiliations:** 1 Department of Pharmacology, University of the Free State, P. O. Box 339 (G6), Bloemfontein 9300, South Africa

**Keywords:** Clinical pharmacology, Developing countries, Trend, Clinical pharmacologist, Research, IUPHAR and World Health Organisation

## Abstract

**Background:**

Several international forums for promoting clinical pharmacology in developing countries have been held since 1980, and several clinical pharmacology programmes targeting developing countries were instituted such that the status of clinical pharmacology in developing countries is not where it was 50 years ago. Therefore, a survey and an appraisal of the literature on the current status of clinical pharmacology in developing countries were undertaken with a hope that it would enable development of appropriate strategies for further promotion of clinical pharmacology in these countries.

**Methods:**

First, nine determinants (or enabling factors) for running a successful clinical pharmacology programme were identified, i.e., disease burden, drug situation, economic growth, clinical pharmacology activities, recognition, human capital, government support, international collaboration, and support for traditional/alternative medicines. These factors were then evaluated with regard to their current status in the developing countries that responded to an electronic questionnaire, and their historical perspective, using the literature appraisal. From these, a projected trend was constructed with recommendations on the way forward.

**Results:**

Clinical pharmacology services, research and teaching in developing countries have improved over the past 50 years with over 90% of countries having the appropriate policies for regulation and rational use of medicines in place. Unfortunately, policy implementation remains a challenge, owing to a worsening disease burden and drug situation, versus fewer clinical pharmacologists and other competing priorities for the national budgets. This has led to a preference for training ‘a physician clinical pharmacologist’ in programmes emphasizing local relevancy and for a shorter time, and the training of other professionals in therapeutics for endemic diseases (task shifting), as the most promising strategies of ensuring rational use of medicines.

**Conclusion:**

Clinical pharmacology in developing countries is advancing in a different way to that in the developed world and continuing support for these efforts will go a long way in promoting improved health for all.

## Background

The need for special focus on clinical pharmacology in developing countries was expressed in several workshops at the first World Congress of Clinical Pharmacology in 1980, and later by Fraser in 1981 [1,2]. Soon after, another international forum aimed at promoting clinical pharmacology in developing countries was held in 1984 under the auspices of the IUPHAR and the Clinical Section of the British Pharmacological Society [3]. This was followed by several communications as well as physical meetings by different stakeholders including IUPHAR and the World Health Organisation (WHO). Wide ranging proposals were made with regard to training, research and service in clinical pharmacology in developing countries by the developed world, including private industry. Since then, several clinical pharmacology programmes targeting developing countries have been instituted [4-8]. These clinical pharmacology programmes were augmented by developments in other sectors particularly economic growth that enabled the construction of essential facilities such as medical schools. These institutions have been the major focus of collaborative programmes on clinical pharmacology by the international community, and many graduates from these institutions have used them as spring boards to further training in clinical pharmacology in the developed countries. Unfortunately, despite such investment, reports on clinical pharmacology in developing countries over the past 50 years have been characterized by the same tone, gloomy: i.e., the discipline still remains in infancy, and that the need for clinical pharmacology here is bigger than anywhere else [1-3,9-11].

In response to such reports, the IUPHAR’s division of clinical pharmacology, through its subcommittee on ‘clinical pharmacology in the developing countries’, embarked on a mission to make visible progress in the development of clinical pharmacology in these countries. Accordingly, the status of clinical pharmacology in developing countries was a subject of a focus conference at the World Congress of Pharmacology 2010 in Copenhagen, in which various speakers expressed their opinions. Again, it was clear that the status of clinical pharmacology in developing countries was not where it was 50 years ago, and that its development had not followed the same path as in the developed world. Later, in its subsequent meeting, the same IUPHAR subcommittee expressed the need for an accurate report on the current status of clinical pharmacology in developing countries, as a pre-requisite to development of appropriate strategies for promotion of clinical pharmacology in these countries.

Unfortunately, most of the information on clinical pharmacology in developing countries is not available in the main stream literature. It is contained in different communications, mainly experts’ reports, for organisations such as the WHO, where it has not been associated with clinical pharmacology. Secondly, these reports are often so detailed and address a variety of multidisciplinary issues such that they are often not suitable for publication in a scientific journal. Here is presented a pragmatic report on the current status of clinical pharmacology in developing countries based on information obtained by a survey on clinical pharmacology activities in some of the developing countries, supplemented by a comprehensive appraisal of the literature. It is hoped that this information will enable formulation of appropriate interventions to foster rational use of medicines in the developing countries.

## Methods

Nine factors were identified as the major determinants (or enabling factors) for running a successful clinical pharmacology programme. They were: the disease burden, the drug situation, economic growth, clinical pharmacology activities (training, research and service), recognition of clinical pharmacology, human capital (clinical pharmacologists and affiliated personnel), local or government support, international support/collaboration and support for traditional/alternative medicines. A survey was undertaken to assess the status and/or existence of some of these enabling factors with a hope that their status would form a useful index for measuring the state of clinical pharmacology at any point in time. The study was approved by the Ethics Committee of University of the Free State (ECUFS ref: 148/2013).

This was a one page questionnaire. After a successful piloting in three institutions, the questionnaire was distributed electronically (by e-mail) worldwide to heads of departments of pharmacology with a help of regional volunteers in Asia, L. America and, Eastern Europe and Africa. It took approximately 15–20 min to complete the questionnaire. Developing countries were determined according to the United Nations Development Programme (UNDP) and World Bank classification of countries [12,13].

Respondents were asked whether clinical pharmacology is a recognised specialty in their country, and if yes, to name the certifying body. It also sought to know whether the respective institution had a dedicated clinical pharmacology department or unit, and if so, the number of professionals serving as clinical pharmacologists and their respective qualifications, i.e., the number of pharmacologists with M.B.Ch.B. and B. Pharm. or equivalent, as well as those with a Ph.D. or equivalent. The questionnaire also probed for presence of scientific forums for pharmacologists, such as a pharmacology society or other, and how often it holds conferences. For information on clinical pharmacology services, respondents were asked to indicate the main clinical pharmacology services undertaken at their departments/units by selecting from a given list, i.e., research, undergraduate teaching, research (clinical trials), postgraduate training, pharmacovigilance, drug utilization, therapeutic patient care/consultation, drug policy or drug regulation, poison information service and other. Respondents were asked to indicate the affiliated personnel with whom the clinical pharmacologists worked (staff members of the units/departments), i.e., medical doctors, pharmacists, nurses, laboratory personnel, poison information officers and others. Regarding training, the questionnaire sought for whether the respective clinical pharmacology departments or units undertake undergraduate and postgraduate training in clinical pharmacology, and for the latter, what qualification was awarded to these graduates, i.e., M.Sc., Ph.D., D.M., M. Med, F.C.P., D.Sc., Dip. The questionnaires also asked whether the institution had adequate number of patients and the disease profile to enable adequate training of clinical pharmacologists, and whether the institution had the appropriate drugs to meet its patient requirements. For those countries that had a drug regulatory authority, it sought the opinion as to whether the respondent was satisfied with the effectiveness of the respective drug regulatory authority, and what fraction (percentage) of drugs on market were made locally versus those imported.

Data was captured on an Excel® data sheet, where the responses were coded and summarised as a percentage of respondents that answered a specific question in a similar way. The result was reported for each status of enabling factors for running a successful clinical pharmacology programme that was evaluated, i.e., clinical pharmacology training, recognition of clinical pharmacology, human capital: clinical pharmacologists and affiliated personnel, local support (government) and international support.

### Literature review

Because some aspects of the enabling factors could not reasonably be determined by the survey, particularly the historical perspectives, information from the literature and relevant experts’ reports from agencies such as WHO and UNAIDS (United Nations Programme on HIV/AIDS), were used to supplement the survey. This was also important for determination of ‘trends in clinical pharmacology’.

## Results

Of the 52 institutions approached, there were 21 respondents (40.4%) despite repeated reminders, i.e., Africa (11), Asia (8), Latin America (3) and Easter Europe (0). They comprised of medical schools (12), pharmacy schools (6), contract research organizations (2) and hospitals (1). Fortunately all the responses could be utilised.

**Figure 1:**
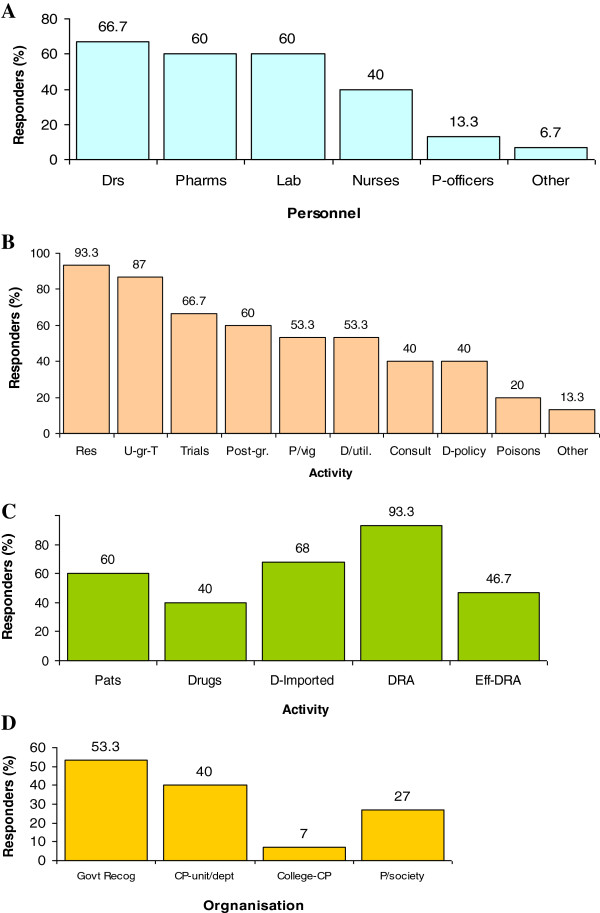
**Enabling factors for clinical pharmacology.** An Illustration of the proportion (%) of respondents regarding: composition of the personnel **(A)**, clinical pharmacology activities **(B)**, drug situation **(C)** and clinical pharmacology recognition **(D)**. Key: A: Drs = medical officers; Lab = laboratory personnel; Other = other health workers. Pharms = pharmacists; P-officers = poison information officers; **B**: Consult = consultations; D/util. = drug utilization; D-policy = drug policy; Other = other services; P/vig = pharmacovigilance; Poisons = poison information services; Post-gr. = post graduate training; Res = research; Trials = clinical trials; U-gra-T = undergraduate training; **C**: D-imported = most drug are imported; DRA = have a drug regulatory authority; Drugs = drugs meet patient requirements; Eff-DRA = ineffective DRA; Pats = had appropriate mix of patients for training purposes; **D**: College-CP = had a College of Clinical pharmacologists; CP-unit/dept = had a clinical pharmacology unit/department; Govt-recog = Clinical pharmacology recognised by government; P/society = had a pharmacology society.

Regarding the professional background of staff members serving as clinical pharmacologists, 34.5% of respondents indicated they had medical graduates (i.e., with M.B.Ch.B. or equivalent), 32.7% had pharmacy graduates, and altogether 38% of the medical and pharmacy graduates had Ph.D. or equivalent. These clinical pharmacologists worked with other personnel in their teams whereby 66.7% of the respondents indicated they had medical officers, 60% had pharmacists, 60% had laboratory personnel, 40% had nurses, and only 13.3% had poison information officers (Figure 1A).

Figure 1B illustrates the on-going clinical pharmacology activities in the study sample. There was wide variation in the clinical pharmacology activities and qualifications offered by different institutions, even from within the same country. Regarding clinical pharmacology services, 40% of respondents undertook bedside patient consultation, 53.3% had a pharmacovigilance programme, 20% offered medical & poison information services, and 40% participated in drug policy formulation or drug regulation. Regarding clinical pharmacology research, the majority (93.3%) of respondents undertook research, but only 66.7% were doing clinical trials (or drug development clinical research), while 53.3% participated in drug utilization studies, and only 13.3% had international collaborative research projects. On clinical pharmacology training, 87% offered undergraduate training, while 60% offered post-graduate training in both basic and/or clinical pharmacology, with only 25% indicating they undertook postgraduate training in clinical pharmacology. Interestingly, there was wide variation in the names or codes used for the postgraduate qualifications in pharmacology and/or clinical pharmacology (M. Sc., Ph.D., D.M., M. Med, F. clinical pharmacology, D.Sc., Dip.), such that it was difficult to determine a basic and clinical pharmacology qualification by the code. In effect, there was no standard qualification for a Clinical Pharmacologist in developing countries.

Figure 1C reflects on the drug situation, whereby only 40% of respondents indicated they had the appropriate number and variety of drugs to meet their patients’ requirements, and that 66% of these drugs were imported. Furthermore, of the 96.3% who indicated that they had a national Drug Regulatory Authority, 46.7% indicated that it was ineffective. These results reflect a situation of chronic drug shortage in the developing countries.

On clinical pharmacology recognition and local support (Figure 1D), 53.3% of respondents indicated that clinical pharmacology is recognised as a specialty by their governments, but, within the institutions, only 40% indicated that they had a dedicated clinical pharmacology division or department. Furthermore, lack of visibility of clinical pharmacology was indicated by the few forums for clinical pharmacology knowledge exchange, whereby only 7% had Colleges of Clinical Pharmacologists and only 27% indicated that clinical pharmacology is part of their national pharmacology society that meets annually.

## Discussion

In general, the survey has articulated the status of the drug situation, clinical pharmacology activities, and extent of recognition and local support for clinical pharmacology in the developing countries. The response rate of 40.4% to the electronic questionnaire was within the expected range of 33.4 ± 9.4% for online surveys, in general [14], and was better than in a previous report on clinical pharmacology in developing countries [15]. Although most of the respondents were from Africa, confining the report to Africa would lead to loss of the contribution by the other half of the respondents, which countries have a lot in common with African countries of similar socio-economic categorization. Furthermore, the categorization of the responding institutions into medical and pharmacy schools, etc., was to emphasize that clinical pharmacologists are not only found in medical schools. Nevertheless, the response rate was still low, as such, the survey data should be regarded as preliminary hoping for confirmation in a larger study. Also, during the preparation of this manuscript, it emerged that the European Association of Clinical Pharmacology and Therapeutics (EACPT) has undertaken a study on clinical pharmacology in Europe but most of this has been communicated only at its congresses.

### Clinical pharmacology activities

Clinical pharmacology activities include clinical pharmacology services, training and research. The scope of these activities is well explained in the recent WHO handbook and the IUPHAR position statement on clinical pharmacology in health care, research and teaching [16,17]. Nevertheless, whereas the two resources are appropriate for understanding the role of clinical pharmacology in health care, research and teaching, they don’t illustrate the scale to which these activities are running, and how much more is needed.

From the survey, it is clear that training and research were the major functions of the clinical pharmacologists in the respondent’s institutions. However, the survey could not determine whether the training was in clinical or basic pharmacology. The challenges of clinical pharmacologists in the developing countries include being trained from overseas in programmes with limited local content or relevancy, lack of appropriate clinical pharmacology training resources that appeal to the conditions in developing countries, limited incentives to clinical pharmacology training, and lack of close partnerships and collaboration among clinical pharmacologists in the developing countries. There is more collaboration with overseas institutions than amongst themselves.

### Human capital

Lack of expertise is one of the biggest contributing factors to the poor clinical pharmacology services in the developing world. Worse still, human capital in any organisation is so dynamic that the results of this 2010-survey may not reflect the reality. Nevertheless, one can confidently say that, even though there was no recognition or accreditation for clinical pharmacologists in most of the developing countries, there is a small pool of clinical pharmacologists working in these countries. There is a need to organise these individuals into a regional or continental professional body that can be used to promote their plight.

### Recognition of clinical pharmacology and local support

Recognition and local support for clinical pharmacology by government refers to the creation of posts and a career path for clinical pharmacologists, while recognition by the host institution gives the discipline a distinction indicated by presence of a clinical pharmacology department or unit in the respective institution. On the other hand, creation of a college of clinical pharmacologists ensures appropriate professional conduct, while forums such as clinical pharmacology society are important not only for sharing scientific information but also for advocacy.

Recognition by governments and professional visibility: Our search showed that by 2007, 84.8% (22/26) of the Eastern Europe countries had successfully recognised clinical pharmacology as a specialty. Furthermore, of the 26 countries in Easter Europe, 14 (50%) had a clinical pharmacology forum or society (Table 1). This was in sharp contrast to Africa where, of the 52 countries, only 5 (9.6%) had a pharmacology society. Even then, South Africa was the only country where clinical pharmacology was recognized as a specialty [18]. Of note, West African countries have opted for a regional society, the West Africa Pharmacology Society, which is dominated by Nigeria and Ghana. In Asia, of the 26 countries, 8 (31%) had a pharmacology society, and only three (Philippines, India and Thailand) had recognized clinical pharmacology as a specialty. In Latin America, of the 18 countries, 7 (39%) had a pharmacology society, and none recognized clinical pharmacology as a specialty. This poor rate of recognition as a specialty was also reflected in the survey.

**Table 1:** Developing countries that had a ‘clinical pharmacology forum’ separate or part of the broad national pharmacology society by 2007

**AFRICA**	**L - AMERICA**	**ASIA**	**E - EUROPE**
Egypt	Argentina	China	Bosnia & Herzegovina
Kenya	Brazil	Indian	Bulgaria
South Africa	Chile	Indonesian	Croatia
	Colombia	Korean	Czech Republic
	Cuba	Pakistan	Estonia
	Venezuela	Philippine	Georgia
	Mexico	Thailand	Hungary
		Malaysian	Latvia
			Lithuania
			Poland
			Romania
			Russia
			Serbia & Montenegro
			Slovakia

**Key**: L-America = Latin America; E-Europe = Eastern Europe.

It must be pointed out that government’s recognition and local support for any speciality precedes the development of ‘human capital’ in that speciality. As such, it is only when a government recognises clinical pharmacology as a speciality that it creates an obligation to meet the training and employment needs of clinical pharmacologists. Unfortunately, although some governments have recognized clinical pharmacology as a specialty, most governments are unwilling to do so owing to the contending priorities that supersede requirements for upcoming specialities such as clinical pharmacology. Nevertheless, appropriate local support should go beyond recognition by way of proclamation. Clinical pharmacology ought to be included in the competing priorities for national and institutional budgets aimed at establishing any facilities for health service, research and training.

### International support

Developing countries have received considerable support from the international community for different purposes, and clinical pharmacology has been one of the main beneficiaries. The support ranges from sponsoring individuals to further their education and training overseas, and funding research capacity strengthening programmes, to direct intervention in running clinical pharmacology services by organisation such as the WHO, USP (United States Pharmacopeia), UNAIDS, to mention but a few. However, the IUPHAR and WHO, through their specialist divisions, remain the major advocates for clinical pharmacology development in the world.

Also, clinical pharmacology training opportunities have been offered in several countries: United Kingdom, United States of America, the Nordic Countries, Belgium, France, Germany, Australia, etc., and most of these have extensive collaborative Research Programmes with the many developing countries; i.e., the North–south collaboration through bilateral agreements, non-governmental organisations and specific institutions, and/or professional societies. The developing countries also benefited from many international private initiatives, specifically philanthropists and other private sponsors, as well as intergovernmental programmes.

As a result, there has been a significant increase in clinical pharmacology activities in many developing countries due to this international collaboration. For instance, in Serbia, there was an increased demand for clinical pharmacology services whereby the number of therapeutic consultations rose from less than 150 per year in 1995, to over 450 in 2003 [19]. From 1995 to 2006, the percentage growths in number of clinical trials was 200% for Africa, 400% for Asia, 800% and 1000% for Latin America [20]. In Russia alone, the number of clinical trials increased from 62 clinical trials run in 315 sites in 2003, to 158 clinical trials run in 863 sites in 2006 [21]. Nevertheless, there is still a need for improvement in clinical pharmacology programmes through increased local and international support.

### The disease burden

The disease burden is a major determinant of the amount and type of medicines required by a given community, and this, in turn, determines the scale of clinical pharmacology services required to ensure rational use of these medicines. From the survey, 60% of respondents indicated that they had adequate number of patients’ disease profile to enable training of clinical pharmacologists (Figure 1D). Although this was a subjective question, the pilot study had shown that respondents understood they were to consider the number of patients and variety of disease conditions in their responses. Nevertheless, 60% is low, and this was most probably because the ‘disease burden’ in these countries is characterised by disease endemics rather than ‘disease-variety’ which the medical school seeks to meet its training requirements.

According to the UNAIDS (2008) report on the global AIDS epidemic, the HIV/AIDS, tuberculosis, malaria, and cardiovascular diseases were the leading causes of morbidity and mortality in the developing world [22]. They were characterized by an increased number of patients without treatment, an urgent need to increase number of patients on treatment, a need to provide lifelong therapy, a need to treat resistant cases and the inadequacy of funding.

Specifically, by end of 2010, there were 2.7 million new cases of HIV world-wide, of which 70% (1.9 million) were from sub-Sahara Africa [23]. Furthermore, the HIV/AIDS-related deaths between 2001 and 2010 increased more than 10-fold in the Eastern Europe and Central Asia, by 60% in the Middle East and North Africa, and more than doubled in East Asia. Regarding the need to treat more patients, only 20% of 34 million people with HIV/AIDS were on treatment. On the other hand, the tuberculosis epidemic posed a serious challenge to the developing world. In 2009, all the 22 high-tuberculosis-burdened countries were from the developing world, and they accounted for over 80% of the new cases of tuberculosis [24]. Worst still, the 27 countries with multi-drug resistant-tuberculosis were from the developing world, and they accounted for 84% of the new cases of multi-drug resistant-tuberculosis. Regarding malaria, in 2010, approximately 81% of the new malaria cases were in Africa and 13% were in the South-East Asia, and 91% of malaria deaths were in Africa [25]. It was estimated that the financial requirement for tuberculosis control is short by $1 billion dollars, while that for malaria is short by $3 billion dollars.

In general, this information shows that the developing world is still characterised by a high disease burden as indicated by several disease epidemics, and this is associated with increased use of medicines with the compounding requirements of rational prescribing and appropriate therapeutic monitoring, all of which require the expertise of a clinical pharmacologist.

### Economic growth

Economic growth was a major determinant of progress in clinical pharmacology development because it enabled establishment of national infrastructures, essential health care facilities, attract and retain expertise, and increased availability of medicines. The best examples are the emerging markets of Eastern Europe where 86% of the countries had recognised clinical pharmacology as a specialty [26].

Since medical schools are in close association with teaching hospitals where most clinical pharmacology programmes are run, a comparison of the number of medical schools in developing and developed countries every 20 years since 1900 was made. This was then related to the level of development of clinical pharmacology programmes in the respective countries or regions. Of note, the WHO defines a medical school as ‘any training institution for health professionals’ and this includes schools of medicine, pharmacy, nursing and allied health sciences.

**Figure 2:**
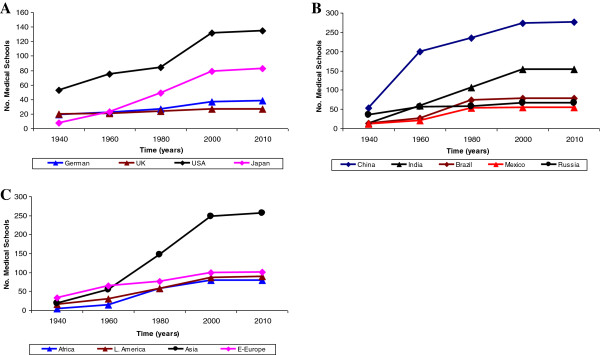
**Medical schools and clinical pharmacology development.** The total number of medical schools every 20 years over the past 100 years in the developed **(A)**, fast developing (Brazil, Russia, India, China and Mexico; **(B)** and least developed **(C)** countries [25].

It was observed that, although presence of a medical school in a country or region was a pre-requisite to the existence of clinical pharmacology, the number of medical schools in a country did not correlate with the level of advancement in clinical pharmacology practice. Clinical pharmacology is well advanced in the United Kingdom, the United States of America and Germany, countries where the number of medical schools has remained consistently low for the past 100 years, and yet, the discipline remained stagnant in developing countries where many new medical schools have been built in the past 50 years (Figure 2).

Specifically, most of the medical schools in the developed countries were built before 1900. By the year 1900, Germany had 19 medical schools, the United Kingdom had 20, the United State of America 53 and Japan 8, and there were virtually no new medical schools after 2000 in each of these countries (Figure 2A). On the contrary, both, the fast developing countries (Brazil, Russia, India and China; BRIC) and the less developed countries, had virtually no medical school before 1900 (Figure 2B and 2C). Medical schools in these countries were built after 1900: viz; in the BRIC countries, 234 new medicals schools were built between 1940 to 1960, and 164 between 1960 to 1980, while in the less developed countries, 174 new medical schools were built between 1960 to 1980 and 176 between 1980 to 2000.

Whereas by 2007 the number of medical schools in Germany, the United Kingdom, the United States of America and Japan increased only to double digits (19, 7, 82 and 75, respectively), the BRIC countries had a total of 629 medical schools (276 in China, 154 in India, 78 in Brazil, 67 in Russia and 54 in Mexico), while the less developed countries had a total of 528 medical schools (257 in Asia [excluding China and India], 101 in Eastern Europe, 90 in Latin America [excluding Brazil and Mexico], and 80 in Africa). Again, it is not the number of medical schools that determine the development of clinical pharmacology, but most probably, the activities within these facilities. This is because these facilities were built to meet other priorities where clinical pharmacology was not included from the onset.

Nevertheless, the developing world exhibited a remarkable increase in the number of medical training facilities in the past 50 years, along with increased clinical pharmacology activities, particularly pharmacology training. This implies that despite the lack of clinical pharmacologists, there are professionals performing such work within these countries. This poses a question as to whether the current definition of a ‘clinical pharmacologist’ which emphasizes the training background of a specialist physician is universally applicable. As such, the claim that clinical pharmacology in the developing countries is still in infancy may be wrong because it ignores the reality that the professionals/practitioners in these countries are the clinical pharmacologists.

### The drug situation

The ‘drug situation’ refers to the extent of utilisation of medicines to combat the disease burden through implementation of appropriate health care and medicines policies. In the developing world, the drug situation is characterised by severe drug shortages due to, among other things, wide spread drug misuse, limited access to drugs due to unaffordable cost and/or unavailability, too many unnecessary drugs, a complex medicine supply system and inadequate information to patients, etc., [27]. This situation was well expressed in the findings of the current survey. Nevertheless, all these problems can be addressed by developing appropriate medicines policies, which includes the policy on the ‘rational use of medicines’.

#### Formulation and implementation of the fundamental policies of health-care

**Figure 3:**
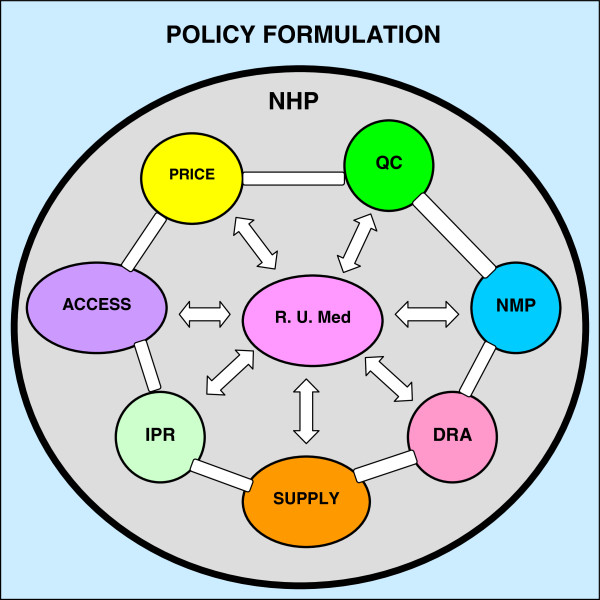
**An illustration of formulation of the policy for rational use of medicines (R.U.Med.) in relation to other health care policies at national levels.** Key: Access = Access policy; DRA = Drug Regulatory Authority; IPR = Intellectual property regulations; NHP = National Health Plan; NMP = National Medicines Policy; PRICE = pricing policy; QC = quality control; Supply = distribution policy.

The major function of clinical pharmacologists is promotion of the rational use of medicines [28,29]. However, because implementation of the policy on rational use of medicines is guided by the national health care policies, clinical pharmacologists need to be part of the teams formulating these policies to ensure that they articulate with the policy on rational use of medicines (Figure 3). Such polices in include: A national health plan, a national medicine policy, a drug regulatory authority, a medicine supply policy, an access to medicine policy, a policy on rational use of medicines, a drug financing or pricing policy, a policy on production and sale of medicines, and a policy on intellectual property rights.

Regarding implementation, Table 2 illustrates the proportion of countries by income group that had established seven of the above mentioned fundamental policies of health-care by 2007. Along side each policy are the respective performance indicators. Overall, 70% to 94% of low income countries had established (written) the health care policies that are fundamental to the development of clinical pharmacology (i.e., a national health plan, a drug regulatory authority, and policies on medicines’ financing, supply, production and sales), and these percentages were not far from the corresponding values of the high income countries. However, the respective performance (or implementation) indicators for these policies in the low income countries lagged behind those of the high income countries. For instance, regarding the national health plan, less than 50% of the low income countries undertook a drug-situation analysis, prescribing audit’ and ‘evaluation of access to health care’. Also, the indicators for implementation of the sub-policies under the drug regulatory authority such as pharmacovigilance (50%) and quality control (68%) were still inadequate. Access to medicines was less than 20% in low income countries versus 75% in high income countries, while the medicines financing policy was only successful for anti-tuberculosis drugs, with research & development scoring an abysmal 16%.

**Table 2:** **Comparison of the proportion (%) of countries that attained a particular policy or indicator in low, mid and high income countries by 2007 **[30]

	**WHO-countries income category**		
**Parameter/Indicator**	**Low income**	**Mid-income**	**High income**
**1. National Medicine Policy (NMP)**	**94%**	**84%**	**74%**
Monitoring: Undertook assessment/audit of:			
• National assessment of NMP	80%	65%	81%
• Pharmaceutical/Drug situation	37%	48%	69%
• Prescriptions audit	39%	46%	71%
• Access	50%	52%	72%
**2. Drug Regulatory Authority (DRA)**	**89%**	**85%**	**97%**
• Marketing authorization of Medicines	88%	84%	94%
• Licensing of manuf., imp./expt. (av.)	91%	91%	99%
• Licensing of prescribers & pharmacies	94%	99%	100%
• Pharmacovigilance (ADR)	50%	64%	67%
• Quality control system in place	68%	69%	96%
**3. Access to essential medicines:** patients within 1-hr walking distance to a clinic, 2003			
• Very low Access (< 50%)	31%	6%	0%
• Low-Medium access (50 – 80%)	56%	38%	25%
• Medium-High access (81-95%)	10%	31%	0%
• Very high Access (< 95%)	2%	25%	75%
**4. Medicine financing and/or pricing policy**	**93%**	**100%**	**100%**
i) With health insurance policy? Medicines received for free:			
• All medicines	35%	59%	55%
• Malaria medicines	59%	72%	47%
• Tuberculosis medicines	100%	92%	94%
ii) Monitoring medicine retail prices:			
• Public sector	40%	58%	77%
• Private sector	36%	49%	78%
• NGO sector	17%	33%	71%
**5. Medicines supply policy**			
• Procurement policy for Ess. Meds	74%	90%	92%
**6. Production and sale of medicines**			
• R&D of new active substances	16%	27%	57%
• Repackaging of finished dos-forms	83%	78%	81%
• National legislation + TRIPS	55%	76%	86%
**7. Rational use of medicines**			

**Key**: *ADR*, adverse drug reactions; *AMR*, antimicrobial resistance; *CME*, continuing medical education; *DTC*, drug therapeutics committees; *Ess. Meds*, essential medicines; *HIC*, high income countries; *LIC*, low income countries; *MIC*, middle income countries; *NGO*, non-government organisation; *NMP*, National medicine policy; *R&D*, research and development; *TRIPS*, trade-related aspects of intellectual property rights.

#### Formulation and implementation of the policy for ‘rational use of medicines’

**Figure 4:**
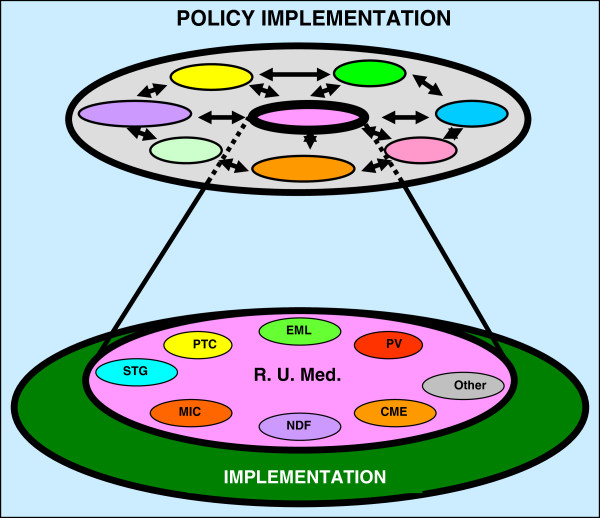
**An illustration of the implementation of the policy for rational use of medicines (R.U.Med.) through its sub-policies at the peripheral level.** Key: CME = Continuing medical education; EML = Essential Medicines List; MIC = Medical and Poison Information centre; NDF = National drug formulary; PTC = Provincial Therapeutic committees; PV = Pharmacovigilance; STG = Standard Treatment Guidelines.

From the afore-mentioned policies, more focused or subject specific policies are drawn to guide policy implemen-tation (Figure 4). In this case, clinical pharmacologists are the leading professionals in the implementation of the policy on ‘rational use of medicines’. This is also emphasized in the WHO recommendations that countries must encourage or ensure more appropriate (rational) use of medicines [29,31] by; establishing a national drug regulatory authority, formulating standard treatment guidelines, selecting an essential medicines’ list, setting up drug or pharmaceutical therapeutics committees, promoting training in good prescribing practices, enforcing continuing medical education, promoting prescribing audit or accredited standard of health care, providing unbiased medical information through medicines information centres and national drug formularies, promoting community education campaigns about medicines and patient package inserts, eliminating perverse financial incentives that lead to irrational prescribing, enforcing ethical medicinal drug promotion or advertising as adopted in resolution WHA 41.17, and advocate for adequate funding for health care.

Regarding implementation, Table 3 shows the proportion of countries by ‘country income group’ that had established nine of the sub-policies for promoting rational use of medicines by 2007. They are also accompanied by the respective indicators for implementation. Whereas the policy documents for promotion of rational use of medicines were available in more than 80% of the low income countries, their respective indicators for implementation were not impressive. Specifically, the proportion of low income countries with programmes for each of the nine indicators illustrated in Table 3 was far lower than that of high income countries. However, some of the respective performance indicators though low, were promising. For instance, the proportion of low income countries with appropriate prescribers at primary health care level, promoting generic substitution, and regulating medicines advertisement were almost similar to that of high income countries (> 85%).

**Table 3:** **Comparison of the proportion (%) of countries that attained a particular policy or indicator for rational use of medicines in low, mid and high income countries in 2007 **[45]

	**WHO-countries income category**		
**Parameter/Indicator**	**Low income**	**Mid-income**	**High income**
**1. Standard Treatment Guidelines (STG)**	89%	75%	80%
**2. Essential Medicines list (EML):**	100%	86%	68%
• EML-Updated in <5 years	81%	74%	41%
**3. Drug Therapeutics Committees (DTC):**	38%	58%	74%
**4. Prescribing policy** or supervision/monitor prescribing			
i) Prescribers at primary care level in public sector			
• Doctors	98%	99%	100%
• Nurses	89%	60%	66%
• Pharmacists	37%	16%	3%
• Other	23%	9%	0%
ii) Policy on generic medicines in public sector			
• Obligatory use of generics	63%	62%	18%
• Generic substitution allowed	85%	87%	77%
• Incentives for prescribing generics	48%	26%	67%
iii) Prescriptions audit	39%	46%	71%
iv) Strategy for AMR containment	24%	46%	73%
**5. Education and Training in CP:** undergraduate and post graduate education (Includes training on EML, STG, pharmacotherapy & Rational prescribing)			
• Doctors	62 ± 3.7%	73 ± 7.1%	88 ± 17.4%
• Nurses	58 ± 10.8	57 ± 3.0%	73 ± 6.0%
• Pharmacists	62 ± 11.5%	60 ± 6.7%	80 ± 13.3%
• Other	33 ± 5.3%	32 ± 6.2%	36 ± 5.3%
**6. Continuing Medical Education (CME):** Obligatory CME for:			
• Doctors	51%	54%	70%
• Nurses + paramedics	53%	44%	65%
• Pharmacists	56%	51%	57%
**7. Provision of information on Medicines:**			
• Med. Information Centres	36%	52%	75%
• Medicines formulary	57%	69%	70%
**8. Public/Consumer education and information on medicines**			
• Education campaigns (A/biotic use)	44%	52%	62%
• Other rational medicine use topics	60%	56%	73%
**9. Medicine promotion & advertising:**			
• Regulate Drug prom/advertisement	85%	86%	100%

**Key**: *AMR*, antimicrobial resistance; *CME*, continuing medical education; *CP*, clinical pharmacology; *DTC*, drug therapeutics committees; *EML*, essential medicines; *HIC*, high income countries; *LIC*, low income countries; *MIC*, middle income countries; *STG*, standard treatment guidelines.

**Figure 5:**
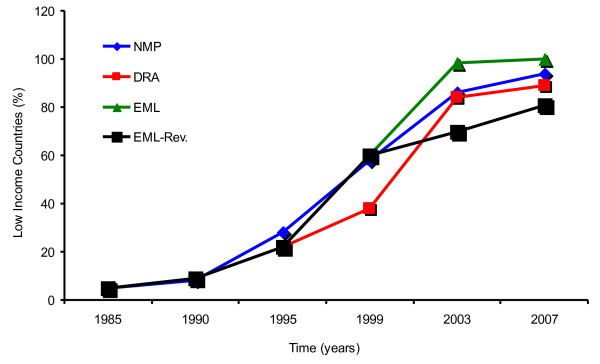
**The proportion (%) of low income countries (LIC) that have formulated the relevant policies from 1985 to 2007 **[30]**.** Key: DRA: Drug Regulatory Authority; EML = Essential Medicines List; EML-Rev. = Essential Medicines List-Revision; NMP National Medicines Policy.

Nevertheless, the poor implementation of the policy on rational prescribing should be expected in view of the fact that implementation of the fundamental health-care policies was also low. The current drug situation in low income countries can be better appreciated when one considers the trend of events over the past 20 or more years. In fact, Figure 5 shows that policy formulation in the low income countries has been a fast process. The proportion of low income countries with a national medicines policy increased from less than 10% in 1985 to 94% in 2007, while the proportion with drug regulatory authorities increased from less than 40% in 1999 to 89% in 2007, and those with an essential medicines list increased from less than 10% in 1985 to 100% in 2007 [32-34]. Likewise, some indicators for policy implementation improved dramatically whereby the proportion of low income countries revising their essential medicines list every five years increased from less than 10% in 1985, to over 80% in 2007 (Figure 5).

Unfortunately, by 2007, the indicators for implementation of sub-policies that ensure rational use of medicines were not different from those of 2003 (Table 4) [31,35]. Whereas the increase in adverse drug reaction reporting (15%) and availability of standard treatment guidelines at national level (22%) were appreciated, these two indicators reflect availability rather than performance. In general, the required policy documents for health-care and rational use of drugs were available in the majority of the developing countries, but their implementation remained poor.

**Table 4:** **Indicators for policy implementation: comparison of the proportion (%) of low income countries that implemented the respective sub-policies for promoting rational use of medicines in 2003 and 2007 **[30]

**Year**	**2003**	**2007**
**Availability of STGs**		
• STGs at National level	67.3%	89%
• STGs at Primary Health level	75%	72%
• NMF for EML	56.4%	57%
**Education and information**		
• EML in Med-Curriculum	67.3%	65.9%
• STG in Med-Curriculum	62.2%	59.5%
• CME for doctors	37.7%	51.1%
• Provided Med. Inform. to prescribers	32.1%	36.2%
• Public Education on Antibiotics	37.0%	44.4%
**Key policies and regulations to promote rational drug use**		
• ADR monitoring	32.7%	50.0%
• DTC in General Hospitals	38.8%	41.5%
• DTC in Regional Hospitals	36.2%	31.4%
• AMR-policy	23.6%	20.0%

**Key**: *ADR*, adverse drug reactions; *AMR*, antimicrobial resistance; *CME*, continuing medical education; *DTC*, drug therapeutics committees; *EML*, essential medicines list; *LIC*, low income countries; *NMF*, national medical formulary; *STG*, standard treatment guidelines.

### Traditional medicines

With the majority of people in developing countries using traditional medicines for their health-care needs and concerns [36-38], there has been a growing recognition of traditional medicines as indicated by a worldwide increase in the number of countries with policies for regulation of traditional medicines, from 15 countries in 1986 to 110 in 2007 [39]. However, several challenges in the regulation of traditional medicines have been encountered. Specifically, the non-standardized classification of traditional medicines has made it more difficult to impose strict rules on their use. Traditional medicines are marketed as herbal medicines, herbal supplements, herbal pharmaceuticals, phytoprotectants or phytotherapeutic agents, or even simply as medicines or as a foodstuff [40]. Also, since one product may be used for several diseases, it is may be sold as a prescription or over-the-counter medicine for self-medication, home remedies, dietary supplements, health foods, functional foods or by some other title [41]. Therefore, depending on the level of sophistication of the regulatory framework, a single medicinal plant may be simultaneously defined and regulated under several different regulatory instruments. The WHO advises that countries must formulate national standards, policies and regulations governing the production and use of traditional medicines to promote and maintain good practice among appropriately-educated producers and practitioners for the benefit of the population [37,42].

Already, several clinical pharmacologists in the developing countries are involved in the establishment of appropriate specifications and standards for traditional medicines that will serve as the basis for consistency, quality control and the verification of safety [43-45], as well as establishment of a post-marketing surveillance system for the evaluation of potential toxicity and herb-herb/herb-drug interactions [32]. For instance, in China, 25% of the medical schools are for Chinese traditional medicines [26], and traditional herbal medicinal preparations constituted between 30% and 50% of the total consumption of medicines in 2005 [39].

### The trend

#### A persistent need for clinical pharmacology

In a nut shell, there are several on-going clinical pharmacology activities in the developing world, but these activities are at different stages in the different countries depending on the level of economic advancement. The persistent epidemics of new and old diseases have led to increased demand for more medicines, which in turn, has increased the requirement for clinical pharmacology services. In the same perspective, the wide scope of clinical pharmacology activities enumerated here may be counter-productive by overstretching and thereby contributing to the scarcity of clinical pharmacologists. This is an appropriate concern, but this report was not on ‘how to utilize clinical pharmacologists’. However, clinical pharmacologists need not be full time employees in some of the roles indicated. In fact, some of the clinical pharmacology activities enumerated here, e.g., drug regulatory authority and national policy formulation, are run by other health care professionals whereby clinical pharmacologists only serve as temporary advisers. Therefore, the use of clinical pharmacologists in some of the clinical pharmacology activities will be determined by the availability of other experts for the respective tasks.

#### Task shifting

**Figure 6:**
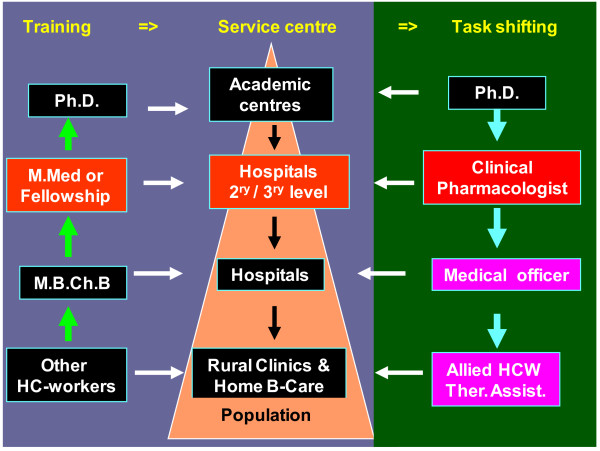
**An illustration of the level of the preferred clinical pharmacologist physician (red box) with regard to level of training (masters), service delivery and task shifting (purple).** Key: 2^ry^/3^ry^ level = Secondary or tertiary level; HCW = Health care workers; HC-workers = Health care workers; Home B-care = Home based care; M.B.Ch.B. = Bachelor of Medicine & Bachelor of surgery; M.Med. = Master of Medicine; Ph.D. = Doctor of philosophy.

Although the number of medical schools do not predict advancement in clinical pharmacology activities, there has been considerable progress in developing clinical pharmacology through training, and running of clinical pharmacology services, particularly by non-governmental organisations such as the WHO and UNAIDS. In the WHO’s programmes and other non-governmental organisations, clinical pharmacologists at their headquarters (located in the developed world) participate in the formulation of the clinical pharmacology policies and programmes, after which these programmes are run by professionals other than clinical pharmacologists, i.e., ‘task shifting’. Specifically, ‘task shifting’ is where some of the allied health professionals (non-medical doctors) are trained in disease-specific therapeutics, e.g., HIV, tuberculosis, etc., to enable them prescribe some medicines under particular circumstances (Figure 6). ‘Task shifting’ is a challenging concept that requires careful planning in the selection, training and monitoring of the workers, patients and the condition in question, but this is beyond the scope for this report. However, success in ‘task shifting’ has been made possible by the fact that, currently, many developing countries have developed and implemented several drug policies and guidelines for promoting rational drug use, which forms the basis for training other professionals.

#### Relevant training

For developing countries, the current training of an internationally recognised clinical pharmacologists with a Ph.D. takes too long. Such training often veers off the local requirements, and ventures into irrelevancy by emphasising highly specialised research over service delivery. Clinical pharmacology training for the developing countries needs to be modified to address local relevancy and within the optimum time. Therefore, it is no wonder that, currently, several developing countries have turned away from doctoral studies as the primary qualification for a clinical pharmacologist, to producing a physician in clinical pharmacology, whereby, after the primary medical degree (M.B.Ch.B.) and at least two years of practice, the candidate undertakes a four year training programme in clinical pharmacology, and graduates with a Masters’ degree (M. Med.) or fellowship in clinical pharmacology (Figure 6). These professionals are employed in tertiary and peripheral hospitals where the government has created posts along side those of other clinical specialities. Even then, this does mean that doctoral studies are irrelevant. Therefore, as for other clinical specialties, these specialist clinical pharmacologists form a pool from which Ph.D. aspirants can be selected.

#### Advocacy

There is a need to promote recognition of clinical pharmacology by high advocacy both locally and internationally, particularly by intensifying IUPHAR’s programmes in this regard. Intervention strategies need to take the stakeholders’ interests into account so as to ensure cooperation. For instance, more government support is likely if the focus is on service based clinical pharmacologists, while international support is more likely with advanced training usually doctoral or research fellowship studies. Clinical pharmacology services should be extended to peripheral hospitals, and play a leading role in the formulation and implementation of drug policies. Besides clinical pharmacology consultation, the afore mentioned clinical pharmacology physicians should also provide special courses in clinical pharmacology (or rational prescribing) for medical students and other health workers as part of the continuing medical education.

## Conclusion

Circumstances in the developing countries foster promotion of ‘a specialist clinical pharmacologist’ and ‘task shifting’ for some endemic diseases as the most appropriate strategies to meet the current and future challenges to rational use of medicines in these countries. This implies that clinical pharmacology in developing countries is advancing in a different way to that in the developed world and that this does not, in any way, mean poor quality or no progress. Continuing support for these efforts will go a long way in promoting improved health for all.

## Competing interests

The author declares that he had no competing interests.

## Authors’ information

Prof. Andrew Walubo M.B.Ch.B., M. Phil., M.D., MBA, FCP. Prof. Walubo is a chief clinical pharmacologist at Universitas academic hospital, and head of department of pharmacology of the University of the Free State. He completed his medical degree at Makerere University in Uganda, master’s studies in Hong Kong, doctoral studies at the University of Cape Town and a postdoctoral fellowship in the Division of Clinical Pharmacology at Vanderbilt University USA.

Prof. Walubo has been instrumental in promotion of clinical pharmacology for a long time. He is a fellow of the American College of Clinical Pharmacology, and founder and ‘Associate Member’ of the ‘The college of Clinical Pharmacologists of South Africa (CCP SA) which governs the training and examination of clinical pharmacology specialists in South Africa. He is a member of the Senate of the Colleges of Medicine of South Africa (CMSA), the Medicines Control Council (MCC; South Africa’s drug regulatory authority) and the Executive committee of the South African Society for Basic and Clinical Pharmacology (SASBCP), to mention but a few. He is a co-author to the 2012 World Health Organization’s (WHO) publication on ‘Clinical Pharmacology in Health Care, Teaching and Research (http://www.who.int/medicines/areas/quality_safety/safety_efficacy/OMS-CIOMS-Report-20120913v4.pdf); to the IUPHAR ‘position statement on Clinical Pharmacology’ in 2010 (Basic Clin Pharmacol Toxicol 2010, 107: 531–559); and a leading author to the recent article (S. Afr.Med.J. 2013;103 (3):150–151) entitled ‘Clinical pharmacology becomes a specialty in South Africa’. Among other things, Andrew is involved in assisting/organising clinical pharmacologists in some countries in Africa.

## Pre-publication history

The pre-publication history for this paper can be accessed here:

http://www.biomedcentral.com/2050-6511/14/49/prepub
